# Ongoing measles outbreak in Wallonia, Belgium, December 2016 to March 2017: characteristics and challenges

**DOI:** 10.2807/1560-7917.ES.2017.22.17.30524

**Published:** 2017-04-27

**Authors:** Tine Grammens, Carole Schirvel, Sylvie Leenen, Nathalie Shodu, Veronik Hutse, Elise Mendes da Costa, Martine Sabbe

**Affiliations:** 1 Service of Epidemiology of Infectious Diseases, Department of Public Health and Surveillance, Scientific Institute of Public Health, Brussels, Belgium; 2 Infectious Disease Surveillance Unit, Agence pour une Vie de Qualité (AVIQ), Walloon region, Charleroi, Belgium; 3 National Reference Centre for measles, mumps and rubella, Service of Viral Diseases, Scientific Institute of Public Health, Brussels, Belgium

**Keywords:** Belgium, Europe, healthcare-associated infections, measles, infection control, post-exposure prophylaxis, public health policy, surveillance, vaccines and immunisation, epidemiology

## Abstract

We describe characteristics of an ongoing measles outbreak in Wallonia, Belgium, and difficulties in control measures implementation. As at 12 March 2017, 177 measles cases were notified, of which 50% were 15 years and older, 49% female. Atypical clinical presentation and severe complications, mainly among adults, in combination with late notification, low or unknown vaccination coverage of contacts, infected healthcare workers and increased workload due to contact tracing, are the main concerns for outbreak management.

Following the detection of a cluster of three measles cases in December 2016, since mid-January 2017, an increasing number of measles cases have been notified in Wallonia, Belgium. Between 20 December 2016 and 16 April 2017, 288 measles cases were reported to the Wallonian regional health authorities [1], compared with 19, 34, 10 and 14 cases in total for 2016, 2015, 2014 and 2013, respectively. We describe the main challenges in the outbreak management such as atypical clinical presentations and difficulties encountered during contact tracing and control measures implementation. As the investigation is still ongoing, we present preliminary findings until 12 March 2017.

Data collection methods and case definitions were described previously [2]. Briefly, cases were classified as possible, probable or confirmed depending on clinical criteria, epidemiological link and laboratory criteria following the case definition of the European Union (EU) Commission Decision of 2012 [3] and an outbreak was defined as two or more laboratory-confirmed cases which are related in time (with dates of rash onset occurring between 7 and 18 days apart) and have epidemiological and/or virological links [4].

## Outbreak description

**Figure 1:**
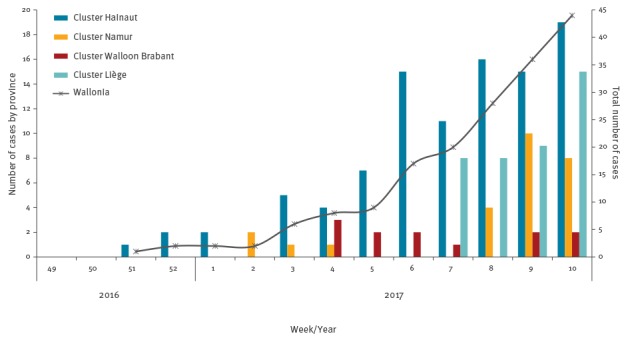
Number of measles cases by week of notification and by province and total number of cases by week of notification, Belgium, 20 December 2016–12 March 2017 (n=177)

The outbreak started as a cluster of three cases notified on 20 December 2016. The 2016 index case was a Belgian resident who had travelled to Romania during the incubation period and this case was most probably imported [5]. Further cases related to the December cluster were mainly notified after mid-January 2017 (Figure 1). Since mid-February 2017, the number of weekly notifications increased considerably, with an average of 36 new measles cases reported per week since week 8 (Figure 1). As at 16 April 2017, there were 288 cases reported; we present data about 177 cases (reported until 12 March 2017) for whom clinical information was collected and recorded.

**Figure 2:**
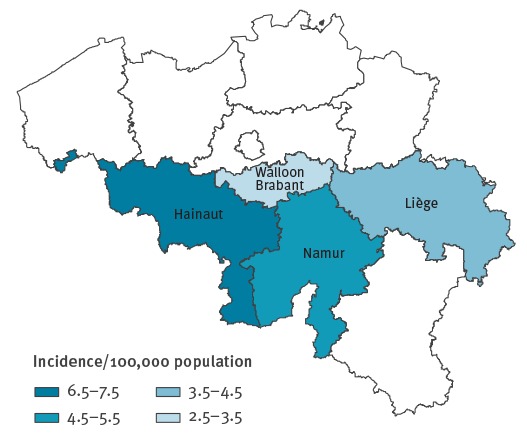
Geographical distribution of measles cases by province, Wallonia, Belgium, 20 December 2016–12 March 2017 (n = 175)

The outbreak has affected four of the five Wallonian provinces: Hainaut (97 cases, 55%), Liège (40 cases, 23%), Namur (26 cases, 15%) and Walloon Brabant (12 cases, 7%) and for two cases location was not reported (Figure 2). The least densely populated province Luxembourg was not affected. The epidemic started in Hainaut in week 3 (5 cases) with rapid transmission from week 6 (15 cases) onwards. The affected patients were mainly of central and eastern European origin, many of them were unvaccinated or had unknown vaccination status, and transmission occurred within families. In the second week of 2017, additional cases occurred in the province of Namur. The cluster resulted in minimum seven nosocomial cases. A third cluster starting in a daycare centre for children between 0 and 3 years of age was notified in week 4 in Walloon Brabant, affected two children (aged 1 and 2 years) and a pregnant woman. In the province of Liège, a hospitalised patient, who had also been in Romania during the incubation period, is suspected to be the source case in another cluster.

Population according to Statistics Belgium 2016 [25].

## Characteristics of cases and vaccination status

**Figure 3:**
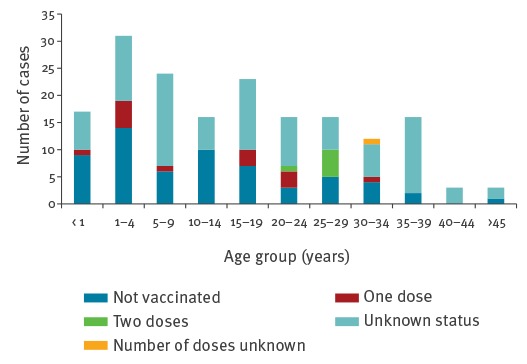
Vaccination status by age group of reported measles cases in Wallonia, Belgium, 20 December 2016–12 March 2017 (n = 177)

Cases were between 5 months and 52 years old, the median age was 14 years. Seventeen cases (10%) were infants under 1 year of age, 31 cases (17%) were 1–4 years, 24 cases (14%) 5–9 years, 16 cases (9%) 10–14 years and 89 cases (50%) were 15 years and older (Figure 3). Eighteen cases (10%) were healthcare workers (HCWs). The majority of cases were not vaccinated (61 cases, 35%) or did not know their vaccination status (95 cases, 54%). Six cases (3%) were reported to be vaccinated with two doses and 15 (8%) with one dose. The M:F ratio was 1.1.

## Clinical presentation and severity

Seventy-six cases (43%) were known to have been hospitalised. Information on reasons for hospitalisation was available for 42 patients, unknown for 32 and registered without complications for two cases. Of the cases with complications, 10 were aged 0–4 years, seven were 5–14 years and 25 were 15 years and older. The main complications in children 0–4 years were: dehydration (n=6), febrile convulsions (n=1), pneumonia (n=3); in 5–14 years old: dehydration (n=4), hepatic cytolysis (n=1), gastro-intestinal problems (n=1) and otitis media (n=1); in adolescents and adults 15 years and older: dehydration (n=6), hepatic disorder and hepatitis (n=8), pneumonia (n=4). One case of acute encephalitis occurred in a young adult 20–30 years old. Other complications in adults were pancreatitis (1 case) and uveitis (1 case). Four pregnant women were confirmed with measles and hospitalised. One pregnant woman developed hepatitis and another had pulmonary complications and preterm delivery. Dehydration in both children and adults was often caused by stomatitis making it difficult to drink. No deaths were reported.

Cases did not always present with the classic triad of symptoms following the EU case definition [3]. Especially among vaccinated persons, fever or rash was sometimes absent, or symptoms appeared in an unusual order (e. g. fever and rash appearing on the same day with no other symptoms). Two vaccinated cases (confirmed by vaccination card) presented only with rhinitis but without rash. Presence of measles virus was however confirmed by PCR. These cases were identified through contact tracing. Some cases were initially not suspected to be measles since symptoms at first presentation were complications such as hepatitis, pancreatitis, pneumonia or stomatitis.

## Laboratory confirmation

As at 12 March, 96 cases were laboratory-confirmed (54%), the majority by the National reference centre (NRC) for measles, mumps and rubella at the Scientific Institute of Public Health (WIV-ISP), 52 were probable cases with epidemiological link to a confirmed measles case, and 29 were possible cases based on clinical picture only.

Genotyping was performed by the NRC. As at publication date, all genotyped cases (n = 44) were classified as B3. All these cases were sequenced and identical to each other and to the strain identified in the December 2016 index case and to the strains circulating in Romania, Italy and Austria at the end of 2016, according to the World Health Organization (WHO) MeaNS database [6].

## Control measures

The regional health authorities in Wallonia have responded to the outbreak according to their guidelines [7] and based on experience of previous years [2]: contact tracing and source investigation was done for each case, cases were isolated where appropriate (e.g. waiting rooms, exclusion from school) and vaccination was proposed to all susceptible contacts through their general practitioners (GPs), paediatricians, HCWs at hospital or occupational medicine. Susceptibility was verified based on vaccination status and date of birth (those born before 1970 were considered as protected according to the guidelines [7]). Two doses of measles vaccine were recommended to susceptible contacts or a second dose was recommended to those who had been vaccinated only once. Information letters to raise awareness were sent to GPs, hospitals, asylum centres and public services for social wellbeing in Wallonia and Brussels capital region, stressing the importance of early case finding, vaccination and notification. Information letters were sent to all parents of the students attending schools and/or classes where measles cases had been reported and to school directors in the province of Hainaut, the most affected region.

By the end of February 2017, the regional health authorities used large scale communication methods (press release, public website, emails, newsflash, sms, intranet for professionals, GP’s and Hospital Infection Control Teams meetings) informing the general population [8,9] and targeting health professionals [10-12] to raise awareness on the high contagiousness of measles, nosocomial infections and vaccination. A risk assessment with all health authorities was conducted at national level on 22 February, informing all regions in Belgium and raising awareness on the difficulties encountered.

WIV-ISP developed a web-based tool for restricted use by surveillance teams in all regions in Belgium, to provide a daily overview on time-place-person in real-time.

## Discussion

In Belgium, measles vaccination is systematically offered since 1985 (one dose) and since 1995 (two doses) [13]. In Wallonia, vaccination coverage for the first dose of MMR measured at age of 18 to 24 months increased from 82.4% in 1999 to 95.6% in 2015 [13], and coverage for the second dose at the age of 11–12 years was 75.0% in 2016 [14]. Since the last large measles epidemic in Belgium in 2011, small outbreaks have occurred, with an average of 68 cases between 2012 and 2016. Measles continues to be considered endemic in Belgium and elimination targets are not yet reached.

The present measles epidemic in Belgium started slowly with a few cases in December 2016, increasing from mid-January and rapidly progressing from mid-February 2017 onwards. At the start of the epidemic in December–January, regular control measures were taken. However, the socioeconomic context of the affected population impacted on contact tracing and active case finding, despite efforts by the health authorities. They were confronted with an unvaccinated population of central and eastern-European origin, not belonging to Sinti or Roma population, residing in Belgium and living in permanent houses, characterised by frequent travel abroad and movements mainly within Wallonia, having frequent family gatherings, language barriers and rarely attending healthcare facilities. So far, no further comparison can be made with respect to the rest of the population, as information on the origin of the patient was not systematically collected. Some cases presented at hospitals at an early stage, without rash or with severe atypical symptoms, and were not identified early as measles cases; this resulted in nosocomial transmission, including among HCWs.

Implementing control measures in newly identified risk groups needs time to understand the complexity of the community. For example, the availability of a mobile vaccination team and facilitated vaccine access might have been helpful to control the cluster in the province of Hainaut and Liège.

Further on, in all provinces, containment was hampered by multiple factors such as atypical clinical presentation with serious complications and, sub-clinical presentations, mainly among partially vaccinated patients. They facilitated the rapid spread of infections due to delay in diagnosis and notification. Moreover, most clinicians had not seen measles in their clinical practice. Delayed isolation of measles cases in hospital settings led to secondary cases including unvaccinated HCWs, resulting in a very high workload of contact tracing and case finding, since they come in contact with many patients and their relatives, especially at emergency wards. As previously described, HCWs affected by measles represented a major challenge in containing the epidemic [15-18]. Timely messages about the risk of unvaccinated HCWs and nosocomial transmission were sent to the hospital hygiene teams, but a legal framework allowing vaccination of HCWs involving occupation health medicine, would be of value.

Even if we cannot exclude an under-reporting of non-complicated cases, the proportion of persons (43%) hospitalised and with complications, is high. The general attitude mentioned by regional health authorities in their communication was to maintain as much as possible patients at home, and not to hospitalise, as precautionary measure to avoid further transmission.

In addition to the increased workload, health authorities were confronted with new case management questions, e.g. in pregnant women [19] and very young infants. The age from which measles vaccination (as a post-exposure prophylaxis) should be administered in order to be effective (e.g. children below 9 months or 6 months [20]), and the time until which this vaccination can be offered, raised questions and issues around the recommendations for the administration of immunoglobulins such as when, how (dose and timing) and to whom, when a person should be considered at risk and which exposure is required to justify immunoglobulin administration. Advice was requested to the Belgian Superior Health Council regarding these case management questions and these aspects are currently under discussion.

Vaccination status of adults is often unknown, and the electronic registry in Wallonia, existing since 2014 [21], is still underused. Especially in the case of HCWs and staff in daycare centres, this is of major concern, since no legal framework exists to guarantee staff’s vaccination against measles. In this outbreak, more than half of the cases were aged 15 years and older. Catch-up vaccination campaigns targeting this group have not yet started and might be hampered by the exclusion of the adult population in the current cost-free vaccination scheme in Wallonia. Exceptional measles in adults vaccinated with two doses against measles, but with positive PCR, have occurred, suggesting the need for serological evaluation of the protective immunity for people working in certain circumstances (e.g. paediatric ward, maternity).

Due to vaccination against measles being part of the childhood immunisation schedule, measles has become rare and a large part of the general population, as well as some physicians, seem to have forgotten measles. Therefore, we are confronted with the question on how to effectively raise awareness of the disease and its potential severity and deadly outcome. At the same time, focus must also remain on vaccination and increasing vaccination coverage to reach the target set by the elimination goals [22,23]. According to The Regional Verification Commission for Measles and Rubella Elimination at the WHO Regional Office for Europe, measles elimination was not reached in 14 of the 53 Member States (26%) of the WHO European Region at the end of 2015 [22]. In January–February 2017, 10 EU/EEA countries reported more than double, the number of cases compared to the same period in 2016 [24]. If the elimination goal is to be reached, the vaccination coverage rates with two doses of measles vaccine will have to be increased in a number of countries, including in Belgium. Also, immunisation gaps need to be closed in those who have missed opportunities for vaccination and attention to specific populations with low vaccination coverage is necessary.
